# From hatch to egg grading: monitoring of *Salmonella* shedding in free-range egg production systems

**DOI:** 10.1186/s13567-019-0677-4

**Published:** 2019-07-30

**Authors:** Andrea R. McWhorter, Kapil K. Chousalkar

**Affiliations:** School of Animal and Veterinary Sciences, The University of Adelaide, Roseworthy, Australia

## Abstract

Human cases of salmonellosis are frequently liked with the consumption of contaminated table eggs. Recently, there has been an increase in consumer demand for cage-free eggs precipitating the need for a greater understanding of *Salmonella* dynamics in free-range production systems. A longitudinal study was conducted to determine the points in production where birds are most likely to be exposed to *Salmonella* and where the risk of egg contamination is highest. In this study, two free-range flocks were sampled from hatch to the end of production. At hatch, all chicks were *Salmonella* negative and remained negative during rearing. During production, the proportion of positive samples was low on both farms. *Salmonella* positive samples were detected intermittently for Flock A. Dust, nest box, and egg belt swabs had the highest proportion of positive samples and highest overall loads of *Salmonella*. The egg grading floor was swabbed at different points following the processing of eggs from Flock A. Only the suction cups that handle eggs prior to egg washing tested positive for *Salmonella*. Swabs collected from machinery handling eggs after washing were *Salmonella* negative. During production, positive samples from Flock B were observed at only single time point. Dust has been implicated as a source of *Salmonella* that can lead to flock to flock contamination. Bulk dust samples were collected and tested for *Salmonella*. The proportion of positive dust samples was low and is likely due to physical parameters which are not likely to support the survival of *Salmonella* in the environment.

## Introduction

*Salmonella* in the food supply chain represents a significant public health threat. Non-typhoidal *Salmonella enterica* serotypes have been estimated to cause over 80 million cases of foodborne human gastrointestinal disease worldwide [1]. While many food items can become contaminated with *Salmonella*, raw eggs and foods containing raw eggs are frequently identified as the bacterial source during trace back epidemiological investigation of human salmonellosis [2, 3]. Due to the implementation of strict egg safety regulations, the total number of egg related cases of salmonellosis has been decreasing in the US and UK [4]. In Australia, during the period spanning 2000–2013, however, a steady increase in the number of egg-related salmonellosis has been observed [3, 5, 6].

Multiple different *Salmonella enterica* serotypes can be found in the layer hen farm environment [7]. Globally, however, two serotypes, *Salmonella* (*S.*) Enteriditis and *S.* Typhimurium, are responsible for causing the vast majority of human disease [2, 8]. *S.* Enteriditis has not been widely detected on Australian commercial egg farms and human infection with this serotype has been predominantly linked epidemiologically with overseas travel [9]. *S.* Typhimurium definitive types (DT) are most commonly isolated during outbreak investigation of Australian egg-related cases of salmonellosis [5, 7].

Upon infection with *Salmonella*, adult layer hens typically do not exhibit clinical symptoms of disease [10, 11] which may be potentially attributed to low dose exposures of *Salmonella* in the shed environment. *Salmonella* subsequently establishes a persistent infection and, as a consequence, birds intermittently shed bacteria in their feces over the course of their productive lifetime [11]. This can lead to increased contamination of the farm environment thereby increasing the risk of horizontal contamination of eggs with *Salmonella*. Vertical transmission of *Salmonella* from hen to egg can also occur [12]. This occurs when the oviduct becomes colonized by *Salmonella* and bacteria are deposited in the egg internal contents during development [12]. While vertical transmission has been described for several *Salmonella* serotypes [13], it is most commonly observed for *S.* Enteriditis [12]. In a recent study, egg shells of birds infected experimentally with *S.* Typhimurium were consistently positive for the bacteria but internal contents were negative [10]. This has also been observed during longitudinal study of *S.* Typhimurium on egg farms [14, 15]. These results indicate that infection of egg internal contents by *S.* Typhimurium is uncommon and that horizontal transmission is the predominant mode of transmission to table eggs for this serotype.

Over the past decade, there has been an increase in consumer demand for cage free eggs in Australia and around the world. This is reflected in the increase in the Australian market share of free range eggs from 26.8% in 2008 to 45.38% in 2018 [16, 17]. Thus, understanding the dynamics of *Salmonella* on free-range farms is a critical aspect of controlling the bacteria in this production system. Both longitudinal and cross-sectional studies of *Salmonella* on free-range layer hen farms have been conducted in the US, UK, and Australia [15, 18–21]. These studies were primarily focussed on identifying the factors that contribute to *Salmonella* contamination of eggs during production. As a consequence, the sampling periods commenced following the onset of lay and did not follow flocks through the end of egg production [15, 19].

It is often assumed that pullets become infected at some stage during rear and that there is a spike in shedding in response to stress experienced by birds at the onset of lay. *Salmonella* epidemiology of newly hatched chicks and during rearing however has, to our knowledge, not been studied extensively. Moreover, most longitudinal studies do not follow flocks through the end of egg production. We have conducted a longitudinal study of *Salmonella* prevalence on two free-range layer farms. This study was conducted over a period spanning from day old hatchlings and rearing through till the end of egg production (75–78 weeks of age) and also on the grading floor while eggs were being graded. The primary aim of the study was to determine the time points when a flock may become exposed to *Salmonella*. Samples were also collected at multiple points prior to and following egg washing on the grading floor to investigate the effectiveness of egg washing on reducing *Salmonella* in post-production processing.

*Salmonella* persistence on farm has been implicated in flock to flock contamination [21]. It has been suggested that residual dust can also serve as a potential source of the *Salmonella* [22]. Thus, an additional aim of this study was to investigate properties of dust that may contribute to the persistence of the bacteria on farm.

## Materials and methods

### Farms

Free-range flocks from farms in each of two Australian states were selected for this study. Both farms had a previous history of *Salmonella* infection and volunteered to participate in this study. Both flocks were comprised of the Hyline brown hen breed. Flock A was initially comprised of 28 000 chicks and was reared on litter. At 15 weeks of age, Flock A pullets were transported to the production farm and split into two sheds. The shed that was sampled during this study housed a total of 14 112 birds. Flock B was comprised of 30 000 chicks from rear. Chicks were raised in an aviary style shed until they were 16 weeks of age and then transported to the production farm. Flock A was permitted to range at 25 weeks of age and Flock B at 26 weeks of age. Birds were provided access to the range for a minimum of eight hours a day (weather permitting) and were locked in the shed at night. Both flocks were not vaccinated against *Salmonella*.

### Rearing phase sample collection

The sample size calculations and sampling strategy of rearing sheds was conducted as designed for a previous study [23]. For Flock A, 1 week prior to placement of chicks, 10 litter and 10 dust swabs were collected and tested for *Salmonella* using culture methods. On the day of chick placement, 20 cm^2^ square sections of transport cage paper underneath the chicks (*n* = 20) were collected and processed for *Salmonella* isolation. Additionally, swabs of the transport cages (*n* = 10) as well as the truck interior and exterior (*n* = 10) were collected. During rearing, 10 dust and 10 litter samples were collected from Flock A at six weekly intervals until pullets were transferred to the production farm. Where possible, swabs of wild bird feces were collected from different sites outside the shed.

For Flock B, prior to chick placement, 10 dust swabs and 12 fecal belt samples were collected and tested for *Salmonella*. As for Flock A, on the day of placement, sections of the transport paper underneath the chicks was also collected and processed. Six swabs of the transport truck were also collected. Samples were collected every 6 weeks during the rearing period. At each sampling time point, 10 dust swabs and 40 fecal swabs were collected and tested for *Salmonella*.

### Production phase sampling

The strategy used for collecting samples in the free-range production was as previously described [15]. For both Flocks A and B, swabs (Whirl–Pak “Speci-Sponge”, ThermoScientific) were premoistened with 20 mL of buffered peptone water (BPW) (Oxoid, Australia). Prior to placement of pullets in the production sheds, one square meter (m^2^) area of the floor slats (*n* = 10), egg belt (*n* = 10), nest boxes (*n* = 10), and dust (top of next boxes) (*n* = 10) were swabbed. This swabbing strategy continued every 6 weeks intervals for Flock A and 10 weeks for Flock B over the productive lifespan of both flocks. The difference in sampling interval was in part due to travel logistics. Additionally, farm staff assisted with the sampling of Flock B and their availability also contributed to the difference. Once egg production started, 30 eggs from egg belt and any floor eggs were also collected at each sampling timepoint and processed using culture methods for *Salmonella*.

### Isolation of *Salmonella* from different samples

Ten grams of litter samples from the rearing shed (Farm A) were combined with 100 mL BPW and incubated for 18 h at 37 °C. One hundred microlitre of the BPW culture was added to 10 mL Rappaport Vassiliadis Soya Peptone Broth (RVS; Oxoid, Australia) and incubated at 42 °C for 18 h. A 10 µL bacteriological loop was used to streak the RVS culture on to a Brilliance *Salmonella* agar (Oxoid, Australia) plate and incubated at 37 °C for 18 h.

For swabs, an additional 20 mL of BPW was added to the Whirl Pak bag and were massaged by squeezing for 30 s. Swabs were squeezed and 18–20 mL of the BPW was collected and incubated at 37 °C for 18 h. One hundred microlitre of this culture was then added to 10 mL RVS and incubated at 42 °C for 18 h. A 10 µL bacteriological loop was used to streak the RVS culture on to a Brilliance *Salmonella* agar plate and incubated at 37 °C for 18 h. Floor eggs and eggs collected from the egg belt were placed into sterile resealable bags in groups of three with 10 mL BPW per egg. Eggs were massaged for 90 s and then placed in to 70% ethanol for 90 s and allowed to air dry. To collect egg internal contents, eggs were cracked, and the contents collected into a sterile resealable bag. Egg contents were thoroughly mixed together, and 2 mL were added to 18 mL BPW. The BPW from the egg shell wash and internal contents was collected and processed as described for swabs.

### Enumeration of *Salmonella*

The microdilution tube most probable number method described by Pavic et al. with some modification was used to enumerate *Salmonell*a in samples [24]. Briefly, 1 mL of homogenized samples was placed in to microdilution tubes (SSIbio, USA) and serial tenfold dilutions were prepared in triplicate. One hundred microlitre of each dilution was then added to microdilution tubes containing 900 µL semi-solid RVS medium with the MRSV *Salmonella* selective agent (Oxoid, Australia). Samples were incubated at 42 °C for 18 h. White colour development indicated presumptive positive *Salmonella* growth. A combination of positive and negative microdilution tubes gave the MPN result. MPN/gram was determined using the MPN tables sourced from the FDA Laboratory Methods [25].

### Grading floor sampling

The prevalence of *Salmonella* on grading floor pre- and post- wash was also assessed. Due to logistics of travel and timing of sampling, only Flock A was sampled. On days when eggs from Flock A were processed, six swabs from the egg suction cups (prior to wash) and six swabs of both the crack detector and egg transfer station were collected and processed as described above for isolation and enumeration of *Salmonella*. On this egg grading floor, suction cups were washed in between the egg grading of different flocks.

### PCR characterization of serotype

A single colony was collected from positive Brilliance *Salmonella* agar plates and placed in to 1 mL of brain heart infusion broth. Samples were grown overnight at 37 °C. Samples were spun at 10 000 g for 5 min. The supernatant was removed, and the pellet was resuspended in 200 µL of six percent Chelex (Biorad, Australia) in Tris–EDTA (TE). Samples were incubated at 56 °C for 20 min, vortexed and then incubated at 100 °C for eight minutes. Samples were then incubated on ice for five minutes and stored at −20 °C until ready for use.

A multiplex PCR described by Akiba et al. was used to identify the serotype of the isolates collected during sampling [26]. Isolates were confirmed as *Salmonella* through the amplification of an *InvA* gene fragment (Forward: 5′-AAACCTAAAACCAGCAAAGG-3′; Reverse: 5′-TGTACCGTGGCATGTCTGAG-3′). Primers designed to the *TSR3* (Forward: 5′-TTTACCTCAATGGCGGAACC-3′; Reverse: 5′-CCCAAAAGCTGGGTTAGCAA-3′) region was used to determine if an isolate was *S*. Typhimurium. PCR reactions were conducted in a total volume of 20 µL. Each reaction contained 4 µL 5× MyRed Taq Buffer, 0.5 µm of each forward and reverse primer for *InvA* and *TSR3*, 0.3 units of MyRed Taq Polymerase (Bioline, Australia), and 2 µL DNA. PCR cycling conditions were as follows: 94 °C for two minutes, 95 °C for 30 s and 60 °C for 30 s repeated for 40 cycles, followed by 72 °C for 5 min.

### Physical and bacteriological analysis of dust

Bulk dust samples were collected from two separate free-range farms. One farm had a history of *Salmonella* and the other did not. 250 mL sample containers were filled with dust collected from the tops of nest boxes or the vents above pop holes. Water activity was tested using an AquaLab Pa_w_ Kit (GrainTec, Australia). The little plastic pan was filled so that it was half full and then the reading was taken. Total moisture was measured using a Mettler Toledo moisture analyser model HE53. A minimum of 0.5 g of each sample was used for total moisture. One gram of dust was added to 10 mL of BPW and incubated for 18 h at 37 °C. One hundred microlitre of the BPW mixture was added to 10 mL of RVS and incubated at 42 °C for 18 h. Samples were streaked on to Brilliance *Salmonella* agar. Dust samples were stored sealed and re-tested for water activity, total moisture and culturable *Salmonella* until each sample was negative.

### Statistics

A Kruskal–Wallis test with post hoc Dunn’s multiple comparison was used to analyze overall prevalence and most probable number data.

## Results

### Prevalence of *Salmonella* during rearing

Flock A was reared on the floor and pre-population litter and dust samples all tested negative for *Salmonella*. On the day of population, 20 sections of paper from underneath the chicks were collected; all were *Salmonella* negative. Ten swabs of the transport cage racks and 10 swabs of the delivery truck were also *Salmonella* negative. Swabs of wild bird feces were also collected from concrete pads underneath feed hoppers outside the shed. One out of six was found to be *Salmonella* positive and was determined by PCR to be *S.* Typhimurium. Litter and dust swabs were collected every 6 weeks during rearing and all tested negative for *Salmonella*.

Flock B was reared in an aviary style shed. Prior to shed population, 12 fecal belt swabs and 10 dust samples were collected and were all *Salmonella* negative. During chick placement, 10 sections of chick transport cage paper and six swabs of the transport truck were collected and were also found to be *Salmonella* negative. Flock B was tested every 6 weeks during rearing and all samples were *Salmonella* negative.

### Prevalence and quantification of *Salmonella* during production

Pre-population dust, floor, nest box and egg belt swabs were collected from the free-range production sheds that housed both Flocks A and B and all were *Salmonella* negative. Five swabs of wild bird feces were collected from areas around the outside of the shed that housed Flock A; all were *Salmonella* negative. Flock A birds were transported to the production shed at 15 weeks of age. The first sampling timepoint for the production phase of Flock A was 1-week post population (16 weeks of age). At this timepoint, only one dust sample tested positive for *Salmonella*. Flock A was subsequently sampled every 6 weeks until the end of production. The final collection was at 71 weeks of age.

**Figure 1:**
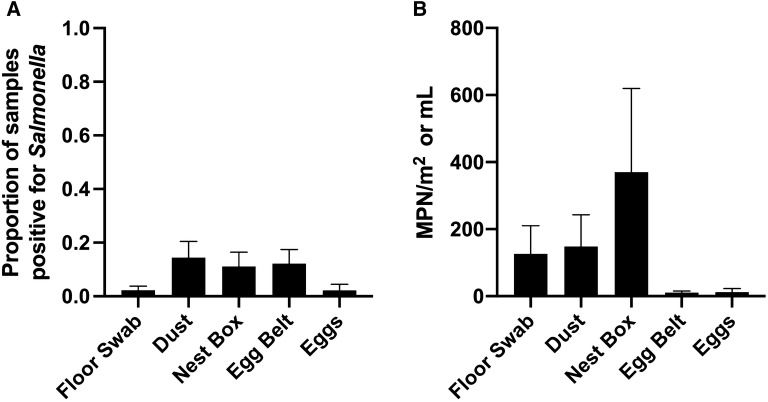
***Salmonella*** **prevalence.** The overall proportion of samples positive for *Salmonella* (**A**). Dust, nest box and egg belts swabs exhibited the highest mean proportion of positive samples, but no significant difference was observed between sample types (*P* > 0.05). Average total *Salmonella* load (**B**) was highest for swabs collected from nest boxes, followed by dust and floor swabs. No statistically significant differences were detected between bacterial loads (*P* > 0.05).

For Flock A, dust, nest box, and egg belt swabs had the highest overall proportion of positive samples (Figure 1A) but were not significantly different from floor swabs or egg shells. The overall load of *Salmonella* (MPN/m^2^) in positive samples was highest for nest box swabs followed by dust and floor swabs (Figure 1B). No significant difference was detected in the total *Salmonella* load across sample type. It should be noted that all floor eggs collected from Flock A over the course of the experiment were negative for *Salmonella*. All egg internal contents were also negative.

For Flock A, the proportion of positive samples was, in general low, but varied over the course of the study. The proportion of positive dust samples ranged from 0.00 ± 0.00 to 0.050 ± 0.17 (Table 1). Dust samples collected at 28 (0.050 ± 0.17) and 52 weeks of age (0.40 ± 0.16) had the highest number of positive samples (Table 1). The total number of positive samples varied significantly over time (*P* < 0.001) but no one sample type had significantly higher proportion of positive samples than another. The greatest proportion of *Salmonella* positive samples was observed at week 28 where a total of 16/50 samples were positive (0.32 ± 0.07). PCR testing revealed that all isolates collected were *S.* Typhimurium.

**Table 1:** **Proportion of** ***Salmonella*** **positive samples and MPN quantification: Flock A**

	Floor	Dust	Nest box	Egg belt	Eggs
16 weeks					
Proportion	0	0.10 ± 0.10	0	0	0
MPN/m^2^	NM	3.0^a^	NM	NM	NM
28 weeks					
Proportion	0.10 ± 0.10	0.50 ± 0.17	0.50 ± 0.17	0.50 ± 0.17	0
MPN/m^2^	43^a^	436.5 ± 237.5	248.4 ± 213.4	9.7 ± 6.7	NM
34 weeks					
Proportion	0	0	0	0	0.20 ± 0.13
MPN/m^2^	NM	NM	NM	NM	12.8 ± 0.4
40 weeks					
Proportion	0	0	0.10 ± 0.10	0.10 ± 0.10	0
MPN/m^2^	NM	NM	3.0^a^	3.0^a^	NM
46 weeks					
Proportion	0	0.10 ± 0.10	0.20 ± 0.13	0.10 ± 0.10	0
MPN/m^2^	NM	4.3^a^	1205 ± 1195	3.0^a^	NM
52 weeks					
Proportion	0	0.40 ± 0.16	0.10 ± 0.10	0.10 ± 0.10	0
MPN/m^2^	NM	3.0 ± 0.0	3^a^	9.2^a^	NM
58 weeks					
Proportion	0	0.10 ± 0.10	0	0.10 ± 0.10	0
MPN/m^2^	NM	9.2^a^	NM	43^a^	NM
64 weeks					
Proportion	0.10 ± 0.10	0	0.10 ± 0.10	0.20 ± 0.10	0
MPN/m^2^	210^a^	NM	43^a^	3.0 ± 0.0	NM
70 weeks					
Proportion	0	0.10 ± 0.10	0	0	0
MPN/m^2^	NM	9.2^a^	NM	NM	NM

Data are presented for each sample type as the proportion of *Salmonella* positive samples (± the standard error of the mean) and most probable number (MPN) (± the standard error of the mean).

^a^Only a single *Salmonella* positive sample was obtained.

The total *Salmonella* load in positive samples was determined using the most probable number method (MPN). The highest bacterial loads were observed in dust swabs at 28 weeks and nest box swabs at 46 weeks with mean MPN/m^2^ of 436.5 ± 237.5 and 1205 ± 1195 MPN/m^2^ respectively (Table 1). At most sampling time points, only a single swab from a particular sample type was positive, as such no statistical analyses could be performed.

The shed that housed Flock B was also swabbed prior to population and all samples were *Salmonella* negative. Birds were moved into the production shed at 18 weeks of age. The first sampling was conducted once the shed had been completely populated (18 weeks of age). Subsequent samplings were conducted at 26, 35, 51, 63 and 68 weeks of age. Differences in sampling regimens were due to interstate travel logistics. *Salmonella* positive samples were observed only at 26 weeks of age. The proportion of positive samples and total *Salmonella* load are shown in Table 2. *Salmonella* isolates collected from positive samples all tested positive for *S.* Typhimurium by PCR.

**Table 2:** **Proportion of** ***Salmonella*** **positive samples and MPN quantification: Flock B**

	Floor	Dust	Nest box	Egg belt	Eggs
26 weeks					
Proportion	0.10 ± 0.10	0.3 ± 0.15	0	0.2 ± 0.13	0
MPN/m^2^	1090^a^	740.6 ± 348.9	NM	556.5 ± 533.5	NM

Data are presented for each sample type as the proportion of *Salmonella* positive samples (± the standard error of the mean) and most probable number (MPN) (± the standard error of the mean).

^a^Only a single *Salmonella* positive sample was obtained.

### Prevalence of *Salmonella* on the egg grading floor

The egg grading and processing facility that handled eggs from Flock A was swabbed at four different time points following the completion of egg processing from that farm. One point prior to egg washing (suction cups) and two points (crack detector and egg weighing station) after washing were swabbed for the presence of *Salmonella*. Only the suction cups tested positive for *Salmonella*. The mean proportion of positive samples was 0.42 with a mean MPN/m^2^ of 117.9 ± 109.1. Both post-wash sampling points were *Salmonella* negative at all sampling time points.

### Persistence of *Salmonella* in dust

**Figure 2:**
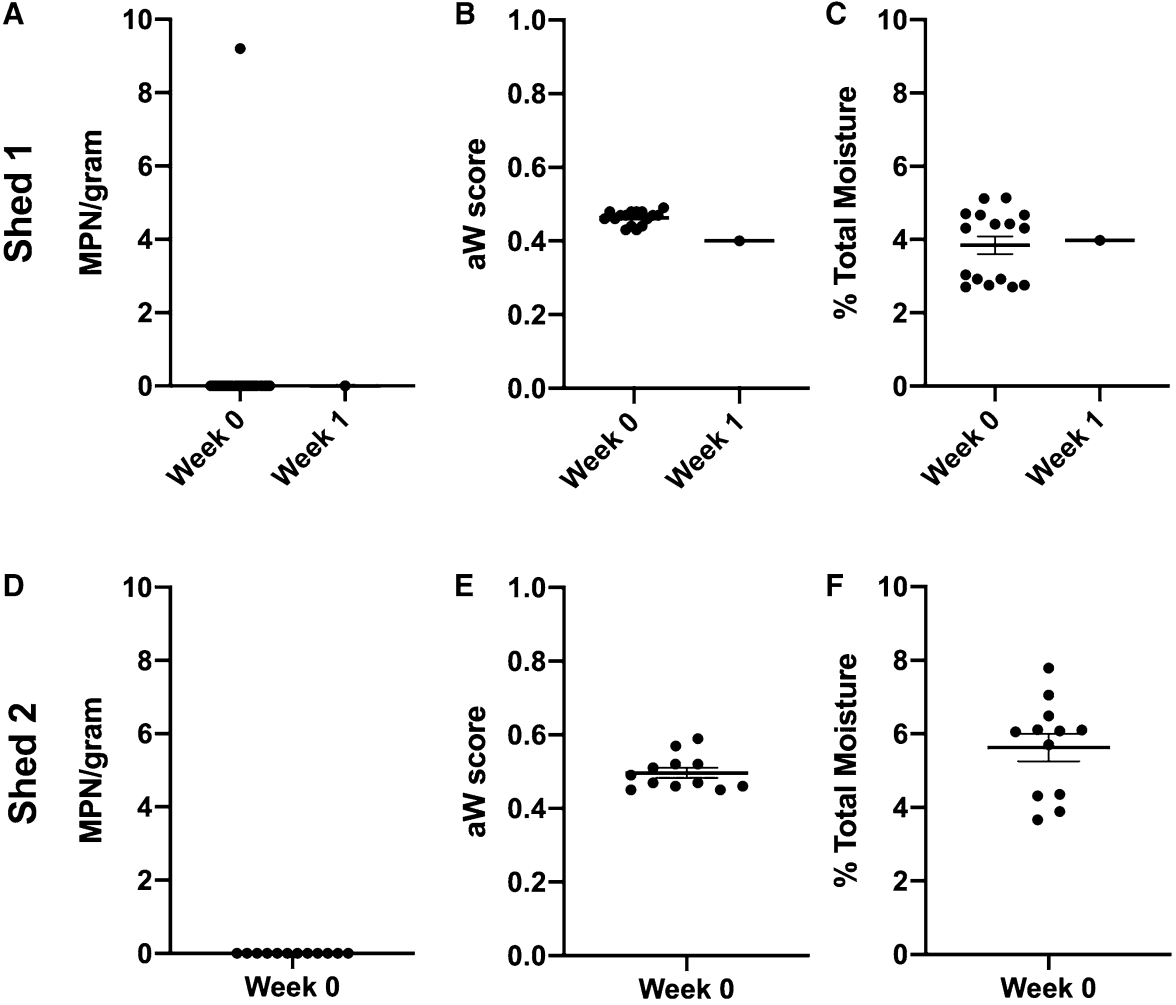
***Salmonella*** **in environmental dust.** Bulk samples of environmental dust was collected from two free-range farms (Shed 1 and Shed 2).Total *Salmonella* load was tested using the MPN method upon collection (**A**, **D**). Only a single sample from Shed 1 was positive for *Salmonella* (**A**). Water activity (**B**, **E**) and total moisture content (**C**, **F**) were measured upon collect for all samples.

It has been suggested that residual dust may serve as a reservoir for *Salmonella* that may facilitate flock to flock transmission of the bacteria. The water activity (a_w_) and total moisture content of dust may be factors that enable the bacteria to persist in the shed environment. Samples were collected from two free range farms. Shed 1 had known history of *Salmonella* infection while Shed 2 had been historically negative. Six samples were collected from each farm at two separate timepoints. One out of 12 dust samples collected from the shed housing Shed 1 tested positive for *Salmonella* enrichment culture (Figure 2A). The total load in this sample was 9.2 MPN/g. Dust from Shed 2 were all negative for *Salmonella* at the time of collection (Figure 2D). One week following the initial collection the one positive sample from Shed 1 was culture negative for *Salmonella*.

Water activity (Figures 2B and E) and total moisture (Figures 2C and F) content of samples was also tested. For Flock A, the mean water activity for samples was 0.47 ± 0.01 a_w_ and mean total moisture content 3.91 ± 0.30% on the day of collection. One-week post collection the one *Salmonella* positive sample had a water activity of 0.40 a_w_ (Figure 2B) and a total moisture content of 3.98% (Figure 2E). For Shed 2, upon collection the mean water activity of the dust samples was 0.53 ± 0.02 a_w_ (Figure 2D). Mean total moisture content was 6.54 ± 0.31% (Figure 2F).

## Discussion

The aim of this study was to follow two free range flocks through their entire productive lifespan (from hatch to end of lay) to characterise critical points where *Salmonella* is likely to be most prevalent. For many Australian egg farms, hatchlings are transported to rearing sheds and raised separately from egg production farms. All chick papers collected from both Flocks A and B were negative for *Salmonella* indicating that upon hatch, chicks had not been infected. To our knowledge, very few field studies have surveyed hatchlings for *Salmonella*. Dust, litter and manure belt swabs were collected for both rearing sheds and B and all were *Salmonella* negative. A single swab of wild bird feces collected from outside the shed housing Flock A was positive for *S.* Typhimurium. Wild bird feces have previously been identified as potential sources of *Salmonella* for free-range flocks [19]. Both Flocks A and B were sampled at 6 and 12 weeks of age. Dust and litter (Flock A) and dust and fecal samples (Flock B) were all negative for *Salmonella*.

In the shed environment, layer hen fecal material, insects, or rodent feces can all be potential sources of *Salmonella* [21, 27]. Prior to placement on the production farms, the sheds that would house Flocks A and B were sampled to characterise the level of residual *Salmonella* post-cleaning. Pre-population samples for both Flocks A and B (dust, floor swab, nest box, and egg belt) were all *Salmonella* negative. Effective cleaning and shed “resting” have been shown contribute to the reduction of *Salmonella* on farm [21, 28]. Both farms in this study house only single age flocks and undergo stringent cleaning protocols between flocks.

When pullets were 15 (Flock A) and 18 (Flock B) weeks of age, they were transferred to the production farms. During the production phase, Flock A was sampled at 6 weekly intervals and Flock B was sampled every 10 weeks. The difference in sampling strategy was due to long distance travel logistics. Over the duration of the study, the total number of positive samples for both flocks did not exceed 20%. Similar other Australian longitudinal studies have reported low *Salmonella* prevalence but the total number of positive samples was higher than the present study [15, 19]. A UK longitudinal study that included sampling on free-range farms, however, reported an overall prevalence of 10.2% which is more consistent with the results presented here [21]. Dust, nest box, and egg belt swabs exhibited the highest overall proportion of positive samples. Chousalkar et al. reported that dust swabs and feces had the highest *Salmonella* prevalence [22]. Eggs collected from the egg belt had a low prevalence of *Salmonella* which is consistent with previous longitudinal studies [15, 19]. Floor eggs collected from both Flocks A and B were all *Salmonella* negative. Gole et al. also tested both floor eggs and eggs collected from the egg belt from multiple free-range farms. They found that the prevalence of *Salmonella* on eggs varied from farm to farm. As with the present study, all eggs on one farm included in their study were all *Salmonella* negative [15]. All internal egg contents were found to be negative for *Salmonella* in this study and is consistent with other studies [15, 19].

Dust, nest box, and floor swabs had the highest bacterial loads from Flock A, while egg belt, dust, and floor swabs positive for *Salmonella* from Flock B exhibited the highest bacterial loads. Gole et al. reported variable bacterial loads in different sample types collected from multiple farms [15]. Longitudinal sampling of another single free-range farm starting from peak lay showed that dust and egg belt samples consistently had high bacterial loads [19].

Egg washing has previously been shown to have significant effects on the reduction of *Salmonella* on the egg shell surface [29]. In this study, egg handling equipment prior to and after egg washing were swabbed for the presence of *Salmonella*. *Salmonella* was detected only on the suction cups prior to washing. All egg handling machinery post-washing were *Salmonella* negative. It should be noted that all egg handling equipment in this facility is disinfected daily and suction cups are changed and disinfected between batches of eggs from different farms. The suction cups were sampled during this study because while grading, cups are exposed to a large number of eggs every day. Instead of testing a large number of eggs from the flock, sampling of suction cups on the grading floor can provide a relatively easy and economical way to understand the positive/negative status of eggs produced by a flock for further testing.

Bulk dust samples were collected from two free-range farms. Samples were tested for the presence of *Salmonella*, total moisture and water activity. Water activity (a_w_) is a measure of the availability of water in a particular medium (e.g. food, dust) and provides a measure of the thermodynamic forces driving the movement of water. Water will tend to move from higher a_w_ to lower a_w_ until an equilibrium is achieved [30]. In general, the total moisture content and water activity of the dust samples collected in this study ranged from 2.7 to 7.8% and 0.40 to 0.59 a_w_ respectively. Both free-range farms use evaporative cooling systems and fans that could affect the overall humidity and moisture content in the shed, so the low water activity and moisture content of the dust sample was initially surprising. The free-range sheds are typically open on average for 8 h per day and given that Australia is a very arid any water in the dust likely moves into the air circulating within the shed. This shed environment may therefore contribute to the inability of *Salmonella* to persist or replicate. A_w_ measurements below 0.6 do not support the culturability or survival of most bacterial species, including *Salmonella* [31]. Future studies are required to determine whether *Salmonella* enters a viable but non-culturable state in dust and whether there are factors that affect the ability of the bacteria to persist.

Epidemiological study of *Salmonella* on free-range farms has, to date, been largely focussed on the egg production period. The present study followed two free-range flocks from hatch to the end of lay. Chicks reared in a “clean” environment remained *Salmonella* negative until they were placed in the production environment. It is likely that birds in Flocks A and B were exposed to extremely low amounts of *Salmonella*. Experimental infection of chicks with a differing amounts of *S.* Enteriditis has demonstrated that at low doses the bacteria is cleared from the liver, a site of persistent infection [32]. It has, however, been demonstrated that persistent infection can be established following a low dose of *S.* Enteriditis and is subsequently shed intermittently in the feces [33]. Models of *Salmonella* transmission in the layer hen environment propose that under farm conditions there is a minimum threshold dose required for the bacteria to establish a persistent infection in a layer hen [34]. This, however, requires further experimental investigation and is likely to be serotype specific.

In summary, the results from this study indicated that the prevalence of *Salmonella* during rear was low, and hence, the risk of *Salmonella* infection during rear appeared to be low. There is always debate on the level of *Salmonella* contamination between cage-free and caged production system. In this study, the level of *Salmonella* in two free range flocks was low compared to previous studies that were conducted in free-range production systems [15, 19]. This suggests that the level of *Salmonella* contamination is dependent on flock and farm management.

## References

[1] Majowicz SE, Musto J, Scallan E, Angulo FJ, Kirk M, O’Brien SJ, Jones TF, Fazil A, Hoekstra RM (2010). The global burden of nontyphoidal *Salmonella* gastroenteritis. Clin Infect Dis.

[2] Threlfall E, Wain J, Peters T, Lane C, De Pinna E, Little C, Wales A, Davies R (2014). Egg-borne infections of humans with *Salmonella*: not only an *S*. Enteritidis problem. World Poultry Sci J.

[3] Moffat CR, Musto J (2013). Salmonella and egg-related outbreaks. Microbiol Aust.

[4] Chousalkar K, Gast R, Martelli F, Pande V (2018). Review of egg-related salmonellosis and reduction strategies in United States, Australia, United Kingdom and New Zealand. Crit Rev Microbiol.

[5] Moffatt CR, Musto J, Pingault N, Miller M, Stafford R, Gregory J, Polkinghorne BG, Kirk MD (2016). *Salmonella* Typhimurium and outbreaks of egg-associated disease in Australia, 2001 to 2011. Foodborne Pathog Dis.

[6] Ford L, Glass K, Veitch M, Wardell R, Polkinghorne B, Dobbins T, Lal A, Kirk MD (2016). Increasing incidence of *Salmonella* in Australia, 2000–2013. PLoS One.

[7] Moffatt CR, Musto J, Pingault N, Combs B, Miller M, Stafford R, Gregory J, Polkinghorne BG, Kirk MD (2017). Recovery of *Salmonella enterica* from Australian layer and processing environments following outbreaks linked to eggs. Foodborne Pathog Dis.

[8] Havelaar AH, Kirk MD, Torgerson PR, Gibb HJ, Hald T, Lake RJ, Praet N, Bellinger DC, De Silva NR, Gargouri N (2015). World Health Organization global estimates and regional comparisons of the burden of foodborne disease in 2010. PLoS Med.

[9] OzFoodNet Working Group (2012) Monitoring the incidence and causes of diseases potentially transmitted by food in Australia: Annual report of the ozfoodnet network, Communicable Diseases Intelligence. pp E13–E241 10.33321/cdi.2018.42.12 30626308

[10] Pande VV, Devon RL, Sharma P, McWhorter AR, Chousalkar KK (2016). Study of *Salmonella* Typhimurium infection in laying hens. Front Microbiol.

[11] McWhorter AR, Chousalkar K (2018). A long-term efficacy trial of a live, attenuated *Salmonella* Typhimurium vaccine in layer hens. Front Microbiol.

[12] Gantois I, Ducatelle R, Pasmans F, Haesebrouck F, Gast R, Humphrey TJ, Van Immerseel F (2009). Mechanisms of egg contamination by *Salmonella* Enteritidis. FEMS Microbiol Rev.

[13] Gast RK, Guard-Bouldin J, Holt PS (2004). Colonization of reproductive organs and internal contamination of eggs after experimental infection of laying hens with *Salmonella* heidelberg and *Salmonella* enteritidis. Avian Dis.

[14] Gole VC, Torok V, Sexton M, Caraguel CG, Chousalkar KK (2014). Association between indoor environmental contamination by *Salmonella enterica* and contamination of eggs on layer farms. J Clin Microbiol.

[15] Gole VC, Woodhouse R, Caraguel C, Moyle T, Rault JL, Sexton M, Chousalkar K (2017). Dynamics of *Salmonella* shedding and welfare of hens in free-range egg production systems. Appl Environ Microbiol.

[16] Australian Eggs Limited (2018) Annual Report 2017/2018, Australian Eggs Limited, North Sydney. New South Wales. https://www.australianeggs.org.au/who-we-are/annual-reports/. Accessed 30 Mar 2019

[17] Australian Eggs Limited (2009) Annual Report: from the farm to the table, Australian Eggs Limited, North Sydney, New South Wales. https://www.australianeggs.org.au/who-we-are/annual-reports/. Accessed on 31 March 2019

[18] Carrique-Mas J, Breslin M, Snow L, McLaren I, Sayers A, Davies R (2009). Persistence and clearance of different *Salmonella* serovars in buildings housing laying hens. Epidemiol Infect.

[19] Chousalkar K, Gole V, Caraguel C, Rault JL (2016). Chasing *Salmonella* Typhimurium in free range egg production system. Vet Microbiol.

[20] Denagamage TN, Patterson P, Wallner-Pendleton E, Trampel D, Shariat N, Dudley EG, Jayarao BM, Kariyawasam S (2016). Longitudinal monitoring of successive commercial layer flocks for *Salmonella enterica* serovar Enteritidis. Foodborne Pathog Dis.

[21] Wales A, Breslin M, Carter B, Sayers R, Davies R (2007). A longitudinal study of environmental *Salmonella* contamination in caged and free-range layer flocks. Avian Pathol.

[22] Chousalkar KK, McWhorter A (2016). Egg production systems and *Salmonella* in Australia. Gast R and Ricke S (edn) Producing Safe Eggs.

[23] Sharma P, Caraguel C, Sexton M, McWhorter A, Underwood G, Holden K, Chousalkar K (2018). Shedding of *Salmonella* Typhimurium in vaccinated and unvaccinated hens during early lay in field conditions: a randomised controlled trial. BMC Microbiol.

[24] Pavic A, Groves P, Bailey G, Cox J (2010). A validated miniaturized MPN method, based on ISO 6579: 2002, for the enumeration of *Salmonella* from poultry matrices. J Appl Microbiol.

[25] Blodgett R (2010) BAM Appendix 2: most probable number from serial dilutions, Bacteriological Analytical Manual, Food and Drug Administration, Silver Spring, MD. https://www.fda.gov/food/foodscienceresearch/laboratorymethods/ucm109656.htm

[26] Akiba M, Kusumoto M, Iwata T (2011). Rapid identification of *Salmonella enterica* serovars, Typhimurium, Choleraesuis, Infantis, Hadar, Enteritidis, Dublin and Gallinarum, by multiplex PCR. J Microbiol Methods.

[27] Davies RH, Breslin M (2003). Persistence of *Salmonella* Enteritidis Phage Type 4 in the environment and arthropod vectors on an empty free-range chicken farm. Environ Microbiol.

[28] Davies R, Breslin M (2003). Observations on *Salmonella* contamination of commercial laying farms before and after cleaning and disinfection. Vet Rec.

[29] Gole VC, Chousalkar KK, Roberts JR, Sexton M, May D, Tan J, Kiermeier A (2014). Effect of egg washing and correlation between eggshell characteristics and egg penetration by various *Salmonella* Typhimurium strains. PLoS One.

[30] Labuza TP, Altunakar B, Barbosa‐Cánovas GV (2007). Fundamental applications. Diffusion and sorption kinetics of water in foods, water activity in foods.

[31] Lenovich LM (2017). Survival and death of microorganisms as influenced by water activity. Rockland (edn) water activity theory and applications to food.

[32] Gast RK, Guraya R, Guard J, Holt PS (2011). Frequency and magnitude of internal organ colonization following exposure of laying hens to different oral doses of *Salmonella* Enteritidis. Int J Poult Sci.

[33] Van Immerseel F, De Buck J, Pasmans F, Bohez L, Boyen F, Haesebrouck F, Ducatelle R (2004). Intermittent long-term shedding and induction of carrier birds after infection of chickens early post-hatch with a low or high dose of *Salmonella* Enteritidis. Poult Sci.

[34] Zongo P, Viet AF, Magal P, Beaumont C (2010). A spatio-temporal model to describe the spread of *Salmonella* within a laying flock. J Theor Biol.

